# Supplementary material for: Milpa Diet for MASLD in Mesoamerican Populations: Feasibility, Advantages, and Future Perspectives
Source: Life (Basel). 2025 May 19;15(5):812. doi: 10.3390/life15050812 (PMC12113525; doi:10.3390/life15050812)
Supplement: Supplementary file 1 [file life-15-00812-s001.zip › life-3530413-supplementary.pdf]

Supplementary Files

Table S1. The effects of milpa diet bioactive compounds in MASLD—the main results of preclinical and clinical studies.

Food	Bioactive Compound	Study Design	Main Results	References
Chili	Capsaicin	Two groups of male Sprague Dawley rats were fed a standard diet and a high-fat diet for 16 weeks. In the high-fat diet group, pelargonic acid vanillylamide was administered for 4 weeks (1 mg/kg/day).	In the rats that received pelargonic acid vanillylamide, there was less progression of fibrosis due to the suppression of the hepatic inflammasome NLRP3 by the downregulation of TLR4/KB and IL-1B.	[83]
		Two groups of 7 male C57/BL6 mice with MASH were each followed for 8 weeks. The intervention group was treated topically with 100 mg capsaicin.	The biopsies showed that capsaicin regulates beta-oxidation and gluconeogenesis through the regulation of the genes carnitine palmitoyltransferase 1 and CD36, respectively, which translates into decreased hepatic steatosis, ALT, and AST levels.	[66]
		Sprague Dawley rats were used. In the intervention group, different doses of capsaicin (0, 2.5, 5.0, or 7.5 mg/kg body weight) were administered intraperitoneally twice daily for 4 weeks.	The higher the capsaicin dose, the greater the reduction in biopsy necrosis and fibrosis, as well as lower hydroxyproline, type III collagen, and hyaluronic acid levels.	[66,84]
		Twelve C57BL/6 mice were fed with a high-fat diet for 20 weeks. Then, they were divided into two groups. The intervention group was supplemented with 0.015% capsaicin.	The intervention group had higher levels of adiponectin. They also had lower levels of free fatty acids, leptin, glucose intolerance, lower weight and smaller adipocytes, TNF-α, and interleukin-6.	[85]
Tomato	Lycopene	Experimental study using two rat models: (1) Sprague Dawley rats fed high-fat diet (HFD, 60% fat) for 12 weeks to induce MASLD, and (2) Zucker diabetic fatty (ZDF) rats (genetic model of obesity/T2DM). Groups received either tomato extract (50–100 mg/kg lycopene) or control.	Tomato extract (100 mg/kg) reduced hepatic steatosis by 42% in HFD Sprague Dawley rats and 38% in ZDF rats vs. controls ($p < 0.01$). Key effects: (1) ↓ hepatic triglycerides (HFD: −47%, ZDF: −39%), (2) ↓ inflammatory markers (TNF-α: −35%, IL-6: −28%), (3) Improved insulin sensitivity (HOMA-IR: −31% in ZDF rats).	[86]
		In total, 40 obese SD rats were fed a high-fat diet for 12 weeks, then divided into control, low-dose (10 mg/kg), and high-dose (20 mg/kg) lycopene groups for 8 weeks. The study measured body weight, lipid profiles, liver enzymes, and hepatic histopathology. It included oral glucose tolerance tests and insulin resistance evaluations.	Lycopene significantly reduced body weight (−18%), improved lipid profiles (LDL ↓ 32%, HDL ↑ 25%), and normalized liver enzymes (ALT/AST ↓ 40%). It reduced hepatic steatosis and inflammation and enhanced insulin sensitivity (HOMA-IR ↓ 35%). Effects were dose-dependent, with high-dose showing optimal protection against MASLD progression.	[87]
		In total, 24 male Sprague Dawley rats were fed standard/high-fat diets ± tomato juice for 5 weeks. The juice contained 10.08 mg/100 g lycopene and key phenolics (chlorogenic acid, rutin). The study analyzed microbiota, SCFAs, and metabolites. A high-fat diet induced grade 2 steatosis.	Tomato juice improved gut health in MASLD rats by increasing Lactobacillus (+11%) and reducing Enterobacteriaceae, while lowering the acetate-to-propionate ratio (−17%). Beneficial phenolic metabolites (3-HPPA, DOPAC) were generated. Despite persistent hepatic fat accumulation, metabolic markers improved.	[88]
Cacao	Epicatechin, catechin, theobromine, caffeine, fiber	Obese male C57BL/6J mice were fed a high-fat (HF) diet for 8 weeks and then randomized to continue HF or HF supplemented with 80 mg/g cocoa powder for 8 more weeks. Hepatic	Cocoa supplementation reduced hepatic triglycerides (28%), lipid peroxidation (56%), and mitochondrial DNA damage (75%). It increased antioxidant enzymes	[68]

		steatosis, oxidative stress, antioxidant enzymes, mitochondrial biogenesis, and SIRT3/PGC1α signaling were analyzed.	and markers of mitochondrial biogenesis via SIRT3/PGC1α activation.	
		Male ZDF (Zucker diabetic fatty) rats (type 2 diabetic model) were fed a 10% cocoa-rich diet (ZDF-Co) or control diet (ZDF-C) for 9 weeks, alongside Zucker lean (ZL) controls. Body weight, glucose tolerance, and insulin sensitivity were monitored. Liver tissue was analyzed for insulin signaling proteins and MAPK (mitogen-activated protein kinase) activation.	Cocoa reduced hyperglycemia, hyperinsulinemia, and weight gain in ZDF rats. It improved insulin sensitivity by lowering IRS-1 (insulin receptor substrate 1) serine phosphorylation, activating the GSK3 (glycogen synthase kinase 3)/glycogen synthase pathway, and modulating gluconeogenic and glycolytic enzymes.	[91]
		Swiss male mice (8-week-old, n = 5/group) fed for 4 weeks with (1) standard diet (N), (2) high-fat diet (HF), (3) cocoa-enriched diet (10% *w/w*, C), or (4) HF + cocoa (HFC). Liver tissues were analyzed for lipid storage, oxidative stress markers, and PPARα signaling.	Cocoa co-administration with HF diet (HFC) reduced hepatic steatosis (↓ 32% triglycerides vs. HF, $p < 0.05$) and normalized weight gain. Mechanistically, cocoa activated PPARα (Peroxisome Proliferator—Activated Receptor Alpha) signaling, restored antioxidant defenses, and prevented HF-induced oxidative stress.	[92]
		A randomized, single-blind, crossover trial in 19 biopsy-proven MASH patients (11M/8F, mean age 46). Participants consumed 40 g/day of dark chocolate (85% cocoa) or milk chocolate (<35% cocoa) for 2 weeks, separated by a 1-week washout. LPS and zonulin levels were measured post-14-day intervention.	Dark chocolate significantly reduced lipopolysaccharide (−15%, $p = 0.04$) and zonulin (−20%, $p = 0.02$) vs. baseline, while milk chocolate had no effect. The lipopolysaccharide reduction correlated with improved gut permeability, possibly through microbiota modulation and NOX2 inhibition (LPS vs. zonulin; R = 0.34, $p = 0.03$).	[89]
		A two-phase study: (1) cross-sectional comparison of 19 MASH, 19 MASLD, and 19 control patients assessing FMD (flow-mediated dilation), NOx (nitric oxide bioavailability), and oxidative stress markers (sNOX2-dp, isoprostanes); (2) randomized crossover trial in MASH patients comparing 14-day intake of 40g/day dark (>85% cocoa) vs. milk chocolate (<35% cocoa) based on the same parameters.	Dark chocolate improved FMD (2.9 ± 2.4% to 7.2 ± 3.0%, $p < 0.001$) and NOx (15.9 ± 3.6 to 20.6 ± 4.9 μM, $p < 0.001$), while reducing oxidative stress. Milk chocolate had no effect. Improvements were correlated with reduced Nox2 (nicotinamide adenine dinucleotide phosphate oxidase isoform 2) activity (Rs = −0.323, $p = 0.04$), suggesting that cocoa polyphenols enhance endothelial function via Nox2 inhibition in MASH.	[90]
Nopal	Betanin, indicaxanthin, piscidic acid, flavonoids	A total of 60 male Wistar rats were fed a high-fat, high-fructose (HFHF) diet for 8 weeks and treated with low/high doses of *Opuntia stricta* peel (OD) or *Opuntia ficus-indica* pulp (OFI) extracts. Oxidative stress, inflammation, DNA damage, and apoptosis markers were analyzed.	OFI (low dose) reduced hepatic triglycerides, while high doses of both extracts improved oxidative stress. H-OFI also lowered inflammation and DNA damage, whereas H-OD reduced apoptosis. OFI was more effective than OD in mitigating steatosis progression.	[93]
		Obese rats were divided into two groups: (1) HFS group (high-fat/high-sucrose diet) and (2) HFS + N group (HFS diet + 5% nopal). After 7 months, the gut microbiota, metabolic endotoxemia (LPS), glucose tolerance, and cognitive function were assessed.	The HFS + N group showed improved gut microbiota (Bacteroides fragilis ↑), reduced metabolic endotoxemia (LPS ↓ 97%), better glucose tolerance, and less hepatic steatosis compared to the HFS group. Nopal also reduced brain oxidative stress and improved cognitive function.	[94]
Cinnamon	Cinnamaldehyde, proanthocyanidins	A total of 48 male Wistar rats fed HFD for 12 weeks received cinnamon (50 or 100 mg/kg/day). Measured metabolic markers, liver enzymes, oxidative stress, inflammation, and gene expression.	Cinnamon improved fatty liver by enhancing fat oxidation (↑ PPARα), reducing fat synthesis (↓ SREBP-1c), and lowering oxidative stress/inflammation. Effects were dose-dependent (100 mg/kg was most effective).	[95]
		Double-blind, placebo-controlled trial; 45 MASLD patients randomized to 1.5 g/day cinnamon or placebo for 12 weeks. Assessed	- Improved insulin sensitivity (↓HOMA, ↑QUICKI).	[70]

		insulin resistance, lipids, liver enzymes, and hs-CRP.	- Reduced fasting glucose, total cholesterol, triglycerides, LDL.<br>- Lowered ALT, AST, GGT, and hs-CRP.<br>- No change in HDL.	
Beans	Fiber, chlrogenic acid, and β-sitosterol	The study induced diabetes in rats using a 2-month high-fat diet and streptozotocin, followed by a 6-week intervention with oat–bean cookies (0.4–1.2 g/kg) or commercial cookies. Serum markers (glucose, lipids, inflammation) and cookie composition (fiber, phytochemicals) were analyzed.	Oat–bean cookies (1.2 g/kg) significantly improved glycemic control (−11% glucose, +20% insulin) and lipid profiles (−28% triglycerides, +74% HDL) in diabetic rats. The atherogenic index and inflammation markers also decreased.	[97]
		An 8-week randomized parallel trial in 26 hypertriglyceridemic women assigned to BOSB (50g/day bean–oat snack bar) or control group.	BOSB group showed: 37.8% ↓ TG (132.04 ± 27.83 vs. 178.87 ± 32.01 mg/dL), 5.69% ↓ glucose (87.55 ± 3.36 mg/dL), and 5% ↓ body weight vs. control.	[96]
Beans and corn	Fiber	The effect of a white corn and bean snack (70/30% blend) was evaluated in mice fed a high-fat diet (HFD) for 12 weeks.	Reduction in weight gain, fat accumulation, adipocyte size, and blood levels of cholesterol, triglycerides, and glucose. An upregulation of the RGS16 gene was observed, which may improve glucose metabolism.	[79]
Maize	Fiber, xylooligosaccharides, ferulic acid, and feruloylated oligosaccharides	Rats were divided into five groups (n = 9 per group). A control group received a standard diet, while the others were administered different combinations of xylooligosaccharides (XOS), ferulic acid (FA), and feruloylated oligosaccharides (FOs) orally for five weeks. Fecal bacterial profiles were analyzed to assess effects on the gut microbiota.	FO treatment significantly increased bacterial richness and enhanced beneficial bacteria (*Actinobacteria, Bacteroides, Lactobacillus*) while reducing *Clostridium* and *Firmicutes*. These microbiota modifications suggest potential for diabetes prevention and weight management by altering disease-associated gut microbial communities.	[98]
Amaranth	High-quality protein, flavonoids, phytosterols, and polyphenols	C57BL/6J mice were fed either (1) a control diet, (2) a high-fat (HF) diet, or (3) an HF diet containing 10% amaranth powder for 8 weeks.	The amaranth group showed (1) significant reductions in hepatic triglycerides, total cholesterol, and phospholipids; (2) the regulated expression of lipogenesis-related genes; and (3) the restoration of gut microbial diversity and richness that had been reduced by the HF diet.	[49]
	Soluble fiber, magnesium, calcium, tocotrienols, and unsaturated fatty acids, especially linoleic acid	Two groups of rats received a standard diet (casein-based) while two others received a standard diet with Amaranthus as the protein source. Within each pair, one group received 20% ethanol in water, creating four experimental groups: CC (casein control), EC (casein+ethanol), CAh (Amaranthus control), and EAh (Amaranthus+ethanol). The estimated ethanol intake was 4–6 g/kg/day per rat.	Amaranthus supplementation prevented serum cholesterol elevation and reduced both free and esterified cholesterol in liver tissue. These effects correlated with the modified expression of lipid metabolism genes (LDL receptor and HMG-CoA reductase). Histological analysis revealed reduced hepatic fat deposits in the Amaranthus groups compared to the casein groups.	[48]
Coriander	Tocopherols, sterols, and carotenoids	Mice with HFD-induced MASLD received oral Coriandrum Sativum L. (CS) extracts (100 or 200 mg/kg/day) for 12 weeks.	CS extract suppressed (1) weight gain, (2) liver weight, and (3) hepatic lipid accumulation by modulating AMPK-SREBP1c (lipid metabolism) and NF-κB (inflammation) pathways.	[99]
Avocado	Vitamin E, lutein, zeaxanthin	Commercial avocado oil (1 mL/250 g body weight) was administered via gavage to MASLD-induced rats. The oil composition was 0.2 g/mL saturated fatty acids (SFAs), 0.2 g/mL polyunsaturated fatty acids (PUFAs), and 0.6 g/mL monounsaturated fatty acids (MUFAs).	Avocado oil treatment (1) improved mitochondrial function, (2) reduced oxidative stress and inflammation, and (3) attenuated MASLD progression by counteracting high-fat/high-fructose-diet-induced mitochondrial and metabolic dysregulation.	[54]
		Diabetic rats received daily avocado oil administration (1 mL/250 g body weight) for 90 days.	Avocado oil improved mitochondrial function and oxidative stress in diabetic livers independently of glycemic control, suggesting potential in mitigating diabetes-associated hepatic damage.	[100]

Insects (grasshoppers)	Protein and fiber	In vivo study that examined the effects of cricket flour on metabolic health in rats fed an obesity-inducing diet.	Supplementing the high-fat, high-fructose diet with cricket flour significantly reduced insulin resistance (lower glucose, insulin, and HOMA-IR levels) and decreased fat accumulation in adipose tissue and the liver. The treatment also improved lipid profiles (reduced triglycerides and LDL, increased HDL) and showed anti-inflammatory effects (lower TNF-α and IL-6).	[101]
Chia	Fiber, omega 3 fatty acids, and antioxidants	Randomized, controlled, and open-label clinical trial (not blinded).<br>Participants: A total of 28 patients diagnosed with MASLD (confirmed by liver elastography). Groups: Chia group, 15 patients received 25 g/day of ground chia seeds. Control group, 13 patients followed a standard diet without supplementation.<br>Duration: 24 weeks.	Supplementation with 25 g/day of chia for six months improved insulin resistance, lipid profiles (increasing HDL), adipose tissue function (↑adiponectin, ↓leptin), and liver parameters (ALT and steatosis). The proposed mechanism behind these benefits is the high content of soluble fiber and alpha-linolenic acid (ALA) in chia, which exert anti-inflammatory and metabolic regulatory effects.	[102]
Garlic	Allicin, diallyl disulfide, S-allylcysteine	C57BL/6J mice fed an HFD (60% kcal fat) for 12 weeks, with daily oral administration of garlic essential oil (25, 50, 100 mg/kg) and diallyl disulfide (10, 20 mg/kg).	Garlic essential oil and diallyl disulfide protected against HFD-induced NAFLD by regulating lipid metabolism, reducing oxidative stress, and suppressing inflammation.	[104]
		Double-blind, randomized, placebo-controlled trial in adults with elevated serum gamma-glutamyl transpeptidase GGT levels who received fermented garlic extract (FGE) daily for a defined period vs. placebo.	FGE significantly reduced serum GGT levels compared to placebo, indicating improved liver function, with less pronounced but potentially beneficial effects on ALT and AST levels. Additionally, the FGE group demonstrated favorable changes in lipid metabolism.	[103]

83. Wikan, N.; Tocharus, J.; Oka, C.; Sivasinprasasn, S.; Chaichompoo, W.; Suksamrarn, A.; Tocharus, C. The capsaicinoid nonivamide suppresses the inflammatory response and attenuates the progression of steatosis in a NAFLD-rat model. *J. Biochem. Mol. Toxicol.* **2023**, *37*, e23279. https://doi.org/10.1002/jbt.23279.
84. Yu, F.X.; Teng, Y.Y.; Zhu, Q.D.; Zhang, Q.Y.; Tang, Y.H. Inhibitory effects of capsaicin on hepatic stellate cells and liver fibrosis. *Biochem. Cell Biol.* **2014**, *92*, 406–412. https://doi.org/10.1139/bcb-2014-0036.
85. Kang, J.H.; Goto, T.; Han, I.S.; Kawada, T.; Kim, Y.M.; Yu, R. Dietary capsaicin reduces obesity-induced insulin resistance and hepatic steatosis in obese mice fed a high-fat diet. *Obesity* **2010**, *18*, 780–787. https://doi.org/10.1038/oby.2009.301.
86. Pipitone, R.M.; Zito, R.; Gambino, G.; Di Maria, G.; Javed, A.; Lupo, G.; Giglia, G., Sardo, P., Ferraro, G., Rappa, F. Red and golden tomato administration improves fat diet-induced hepatic steatosis in rats by modulating HNF4α, Lepr, and GK expression. *Front. Nutr.* **2023**, 10, 1221013.
87. Baz, L.; Algarni, S.; Al-thepyani, M.; Aldairi, A.; Gashlan, H. Lycopene Improves Metabolic Disorders and Liver Injury Induced by a Hight-Fat Diet in Obese Rats. *Molecules* **2022**, *27*, 7736.
88. García-Alonso, F.J.; González-Barrio, R.; Martín-Pozuelo, G.; Hidalgo, N.; Navarro-González, I.; Masuero, D.; Soini, E.; Vrhovsek, U.; Periago, M.J. A study of the prebiotic-like effects of tomato juice consumption in rats with diet-induced non-alcoholic fatty liver disease (NAFLD). *Food Funct.* **2017**, *8*, 3542–3552. https://doi.org/10.1039/c7fo00393e.
89. Pannunzio, A.; Baratta, F.; Maggio, E.; Palumbo, I.M.; Magna, A.; Trivigno, C.; Carnevale, R.; Simona, B.; Cammisotto, V.; Vidili, G.; et al. Dark chocolate's impact on low-grade endotoxemia in metabolic dysfunction-associated steatohepatitis. *Nutrition* **2025**, *131*, 112643. https://doi.org/10.1016/j.nut.2024.112643.

90. Loffredo, L.; Baratta, F.; Ludovica, P.; Battaglia, S.; Carnevale, R.; Nocella, C.; Novo, M.; Pannitteri, G.; Ceci, F.; Angelico, F.; et al. Effects of dark chocolate on endothelial function in patients with non-alcoholic steatohepatitis. *Nutr. Metab. Cardiovasc. Dis. NMCD.* **2017**, *28*, 143–149. https://doi.org/10.1016/j.numecd.2017.10.027.
91. Cordero-Herrera, I.; Martín, M.Á.; Escrivá, F.; Álvarez, C.; Goya, L.; Ramos, S. Cocoa-rich diet ameliorates hepatic insulin resistance by modulating insulin signaling and glucose homeostasis in Zucker diabetic fatty rats. *J. Nutr. Biochem.* **2015**, *26*, 704–712. https://doi.org/10.1016/j.jnutbio.2015.01.009.
92. Fidaleo, M.; Fracassi, A.; Zuorro, A.; Lavecchia, R.; Moreno, S.; Sartori, C. Cocoa protective effects against abnormal fat storage and oxidative stress induced by a high-fat diet involve PPARα signalling activation. *Food Funct.* **2014**, *5*, 2931–2939. https://doi.org/10.1039/c4fo00616j.
93. Besné-Eseverri, I.; Martín, M.Á.; Lobo, G.; Cano, M.P.; Portillo, M.P.; Trepiana, J. Antioxidant and Anti-Inflammatory Effects of Opuntia Extracts on a Model of Diet-Induced Steatosis. *Antioxidants* **2024**, *13*, 1416. https://doi.org/10.3390/antiox13111416.
94. Sánchez-Tapia, M.; Aguilar-López, M.; Pérez-Cruz, C.; Pichardo-Ontiveros, E.; Wang, M.; Donovan, S.M.; Tovar, A.R.; Torres, N. Nopal (*Opuntia ficus indica*) protects from metabolic endotoxemia by modifying gut microbiota in obese rats fed high fat/sucrose diet. *Sci. Rep.* **2017**, *7*, 4716. https://doi.org/10.1038/s41598-017-05096-4.
95. Li, B.; Li, J.; Hu, S. Cinnamon could improve hepatic steatosis caused by a high-fat diet via enhancing hepatic beta-oxidation and inhibiting hepatic lipogenesis, oxidative damage, and inflammation in male rats. *J. Food Biochem.* **2022**, *46*, e14077. https://doi.org/10.1111/jfbc.14077.
96. Ramírez-Jiménez, A.K.; Luzardo-Ocampo, I.; Cuellar-Nuñez, M.L.; Anaya-Loyola, M.A.; León-Galván, M.F.; Loarca-Piña, G. Daily Intake of a Phaseolus vulgaris L. Snack Bar Attenuates Hypertriglyceridemia and Improves Lipid Metabolism-Associated Plasma Proteins in Mexican Women: A Randomized Clinical Trial. *Front. Nutr.* **2022**, *9*, 890136. https://doi.org/10.3389/fnut.2022.890136.
97. Pérez-Ramírez, I.F.; Becerril-Ocampo, L.J.; Reynoso-Camacho, R.; Herrera Guzmán-Maldonado, S.H.; Cruz-Bravo, R.K. Cookies elaborated with oat and common bean flours improved serum markers in diabetic rats. *J. Sci. Food Agric.* **2018**, *98*, 998–1007. https://doi.org/10.1002/jsfa.8548.
98. Ou, J.Y.; Huang, J.Q.; Song, Y.; Yao, S.W.; Peng, X.C.; Wang, M.F.; Ou, S.-Y. Feruloylated Oligosaccharides from Maize Bran Modulated the Gut Microbiota in Rats. *Plant Foods Hum. Nutr.* **2016**, *71*, 123–128. https://doi.org/10.1007/s11130-016-0547-4.
99. Gu, M.J.; Ahn, Y.; Lee, Y.R.; Yoo, G.; Kim, Y.; Choi, I.; Ha, S.K.; Kim, D. *Coriandrum sativum* L. Leaf Extract Ameliorates Metabolic Dysfunction-Associated Steatotic Liver Disease by Modulating the AMPK Pathway in High Fat-Fed C57BL/6 Mice. *Nutrients* **2024**, *16*, 4165. https://doi.org/10.3390/nu16234165.
100. Ortiz-Avila, O.; Esquivel-Martínez, M.; Olmos-Orizaba, B.E.; Saavedra-Molina, A.; Rodriguez-Orozco, A.R.; Cortés-Rojo, C. Avocado Oil Improves Mitochondrial Function and Decreases Oxidative Stress in Brain of Diabetic Rats. *J. Diabetes Res.* **2015**, *2015*, 485759. https://doi.org/10.1155/2015/485759.
101. Escobar-Ortiz, A.; Hernández-Saavedra, D.; Lizardi-Mendoza, J.; Pérez-Ramírez, I.F.; Mora-Izaguirre, O.; Ramos-Gómez, M.; Reynoso-Camacho, R. Consumption of cricket (*Acheta domesticus*) flour decreases insulin resistance and fat accumulation in rats fed with high-fat and -fructose diet. *J. Food Biochem.* **2022**, *46*, e14269.
102. Medina-Urrutia, A.X.; Jorge-Galarza, E.; El Hafidi, M.; Reyes-Barrera, J.; Páez-Arenas, A.; Masso-Rojas, F.A.; Martínez-Sánchez, F.D.; López-Uribe, Á.R.; González-Salazar, M.D.C.; et al. Effect of dietary chia supplementation on glucose metabolism and adipose tissue function markers in non-alcoholic fatty liver disease subjects. *Nutr. Hosp.* **2022**, 39, 1280–1288. https://doi.org/10.20960/nh.04084.
103. Kim, H.N.; Kang, S.G.; Roh, Y.K.; Choi, M.K.; Song, S.W. Efficacy and safety of fermented garlic extract on hepatic function in adults with elevated serum gamma-glutamyl transpeptidase levels: A double-blind, randomized, placebo-controlled trial. *Eur. J. Nutr.* **2017**, *56*, 1993–2002. https://doi.org/10.1007/s00394-016-1318-6.
104. Lai, Y.S.; Chen, W.C.; Ho, C.T.; Lu, K.H.; Lin, S.H.; Tseng, H.C.; Lin, S.-Y.; Sheen, L.-Y. Garlic essential oil protects against obesity-triggered nonalcoholic fatty liver disease through modulation of lipid metabolism and oxidative stress. *J. Agric. Food Chem.* **2014**, *62*, 5897–5906. https://doi.org/10.1021/jf500803c.
